# Expression of Active Apurinic Apyrimidinic Endodeoxyribonuclease 1 in Human Platelets

**DOI:** 10.1096/fj.202603193RR

**Published:** 2026-08-28

**Authors:** Isabella Parolini, Alessia Bellina, Giada Carpi, Evgeniia A. Diatlova, Giovanna Lippe, Elisabetta Fontanini, Giovanni Barillari, Miriam Isola, Antonio P. Beltrami, Gianluca Tell

**Affiliations:** ^1^ Department of Oncology and Molecular Medicine Istituto Superiore di Sanità Rome Italy; ^2^ Department of Medicine University of Udine Udine Italy; ^3^ Institute of Clinical Pathology Azienda Sanitaria Universitaria Friuli Centrale (ASUFC) Udine Italy; ^4^ Center for Hemorrhagic and Thrombotic Diseases General and University Hospital S. Maria Della Misericordia Udine Italy

**Keywords:** activity, APE1, epinephrine, mitochondria, platelet

## Abstract

The pivotal role of platelets (PLTs) in hemostasis, thrombosis, innate immunity, and tissue repair is sustained by mitochondria, whose dysfunctions can impair ATP generation and redox signaling, thus affecting optimal levels of PLTs' functionality. Maintenance of mtDNA integrity is essential for mitochondria functioning and is primarily exerted by the base excision repair (BER) pathway, never described in PLTs. Here, we provide the first evidence for the expression levels and subcellular localization of apurinic/apyrimidinic endodeoxyribonuclease 1 (APE1), evaluated alongside cytosolic and mitochondrial markers, in resting human PLTs (huPLTs) isolated from a cohort of healthy donors. A variable expression of APE1 was detected independently of age and sex, and it was found to correlate with the specific enzymatic activity on canonical and far less on noncanonical substrates. Preliminary experiments with an APE1 enzymatic inhibitor suggest a possible involvement of APE1 in PLTs aggregation stimulated by epinephrine. Overall, our findings pave the way for understanding a novel unforeseen role of APE1 in PLTs physiology, which specifically deserves future investigation.

## Graphical abstract

Platelets (PLTs) isolated from healthy donors contain variable amounts of enzymatically active APE1, likely surveilling mitochondrial DNA integrity and overall mitochondria functioning. The use of the specific APE1 inhibitor compound #3 on platelet rich plasma (PRP) significantly impairs the PLTs' aggregation properties of activated PLTs triggered only in the presence of epinephrine agonist, thus unveiling the ATP‐producing function of mitochondria in the observed phenomena and an important role of APE1 in this pathway and in platelets' physiology.

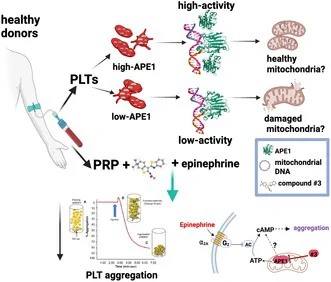

1

Although platelets (PLTs) lack a nucleus and the ability to synthesize new proteins, they are equipped with numerous mitochondria (5–8 per platelet) that play essential roles in metabolism, ATP production, calcium homeostasis, apoptosis, and reactive oxygen species (ROS) signaling—all fundamental processes for PLTs function [1]. Furthermore, these mitochondria also contain their own DNA (mtDNA), which is responsible for coding 13 essential subunits of the oxidative phosphorylation complex (OXPHOS), thereby playing a crucial role in cellular energy conversion [2].

In physiological conditions, the continuous cell exposure to ROS promotes direct bases oxidation on DNA and generates oxidized nucleotides that can be incorporated into DNA, thus inducing base mispairing during DNA replication, in turn leading to genome instability and break‐induced mutations [3]. mtDNA lacks protective histones and its location close to sites of OXPHOS continually exposes it to ROS produced during respiration, thus inducing mutagenic damage that can compromise the function of the respiratory chain generating ATP [4].

As a result, in PLTs the impairment of the energy production can induce premature death and reduced aggregation, thus affecting hemostasis, procoagulant state, and inflammation [5].

To counteract such damages, mitochondria are endowed with a quality control mechanism, based on rapid mtDNA turnover, dynamic fission and targeted mitophagy [6], and a less energetically expensive DNA repair mechanism led by the base excision repair (BER) pathway [7], whose presence has never been investigated in PLTs, yet. In this pathway, an essential role is exerted by apurinic/apyrimidinic endodeoxyribonuclease 1 (APE1), a 35.6 kDa ubiquitous multifaceted protein, that includes two isoforms, a full‐length one (p37) and a N‐terminal truncated form (p33), generated by proteasomal degradation [8]. APE1 recognizes abasic sites, cleaves the phosphodiester backbone at the 5′‐end and allows the faithful repair of the lesion by further downstream enzymes of BER [9]. APE1 is the main abasic endonuclease of mammalian cells. It is principally present in the nucleus of cells, and, in lower amounts, in the mitochondria, where it is imported by Tom‐20 through the interaction of some specific residues (i.e., Lys299, Arg 301) [10]. Importantly, the increase of APE1 in the mitochondrial matrix was found to correlate with a decrease in mtDNA lesions [11].

PLTs are devoid of nuclei and, to date, studies on mtDNA repair mechanisms in PLTs remain limited, with the exception of a recent study describing the role in thrombus formation played by MTH1, an enzyme of the mismatch‐repair pathway, that prevents the misincorporation of oxidized nucleotides in the mtDNA [12]. Assessing APE1 expression and activity in human PLTs (huPLTs) could help elucidating the involvement of the BER pathway in healthy or pathological conditions.

We conducted our study on huPLTs isolated from healthy donors right after blood withdrawn to avoid the PLTs' activation, as depicted in Figure 1A. We enrolled male and female blood donors in good health condition, not taking medications and/or aspirin in the preceding week. To minimize the intra‐individual variability, samples were collected early in the morning after overnight fasting. Right after blood processing, we ensured the quality of PLTs preparation by cytofluorimetric analysis (Figure 1B). Results obtained from the entire cohort enrolled indicated high CD42b positivity (median frequency of positive events = 83%) and a barely detectable expression of CD45 positive events (median frequency of positive events = 0.7%) that, given their size range of about 1 μm (Figure 1B, violin plots), could be related to leukocyte‐derived fragments. Furthermore, the observed differences in CD42b expression may be attributed to variations in PLTs' age across donors since the surface antigenic properties of younger PLTs are distinct from those of mature PLTs [13]. To further confirm the purity of the PLT population, we performed CD45 immuno‐depletion assay followed by FACS analysis and found the absence of contaminating leukocytes in the PLTs population, in line with Figure 1B (data not shown).

**FIGURE 1:**
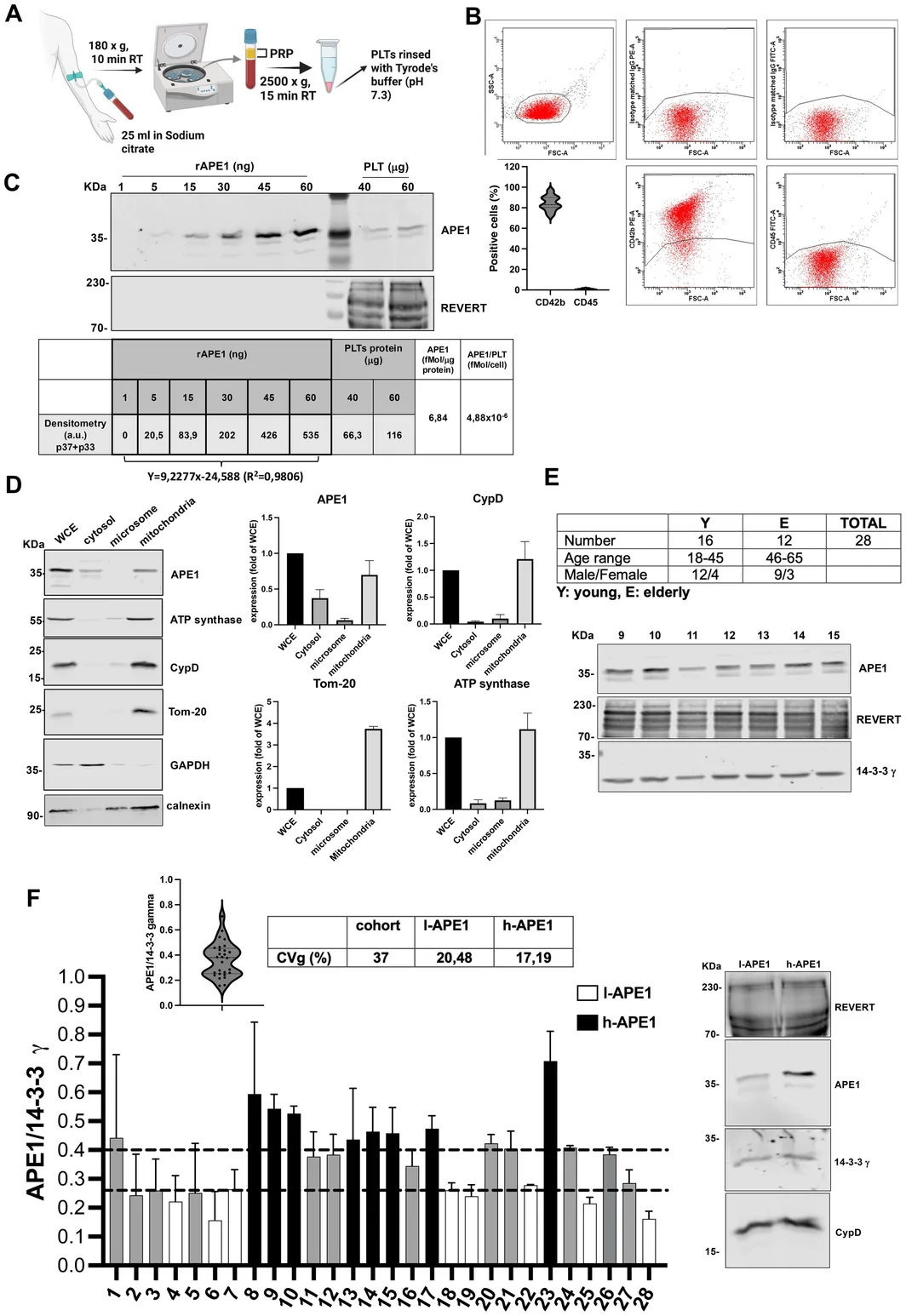
Expression, quantification and distribution of APE1 in huPLTs. Panel (A) Workflow of PLTs isolation from human blood donors. All experimental conditions involving collection of blood from volunteers were approved by the Institutional Review Board (IRB) of the University of Udine (RIF Prot IRB: 047/2025, Til III cl 13 fasc.24/2025), and written informed consent was obtained from each subject. Whole blood (25 mL) was drawn into tubes anticoagulated with trisodium citrate and immediately processed to obtain platelet‐rich plasma (PRP) (5 mL/donor). PLTs were rinsed with Ca^2+^ free‐HEPES‐Tyrode's buffer (HTB; pH 7.3) before further analyses. Created with Biorender.com. Panel (B) Representative flow cytometry analysis of PLTs. Physical parameters are shown (FSC‐A and SSC‐A) for PLTs population labeled with Phycoerythrin‐conjugated (PE)‐CD42b and Fluorescein 5‐isothiocyanate (FITC)‐CD45, and relative isotype controls. The shown gate was set employing 1 μm size calibrating beads. However, all the events were considered for CD42b and CD45 statistics. On the bottom left of the panel, a violin plot with CD42b + and CD45‐ PLTs of the entire cohort indicates the absence of contaminating leukocytes in PLTs preparations. Panel (C) Western blot analysis of known doses of rAPE1 as indicated (ng), and PLTs total lysate (50 μg) from randomized subjects (*n* = 4). PLTs total lysate was obtained through sonication (3 cycle of 5 s sonication and 30 s resting) on ice. The membrane was probed with anti‐APE1 antibody and REVERT was used for total protein staining. The densitometry values reported in the Table were used to generate a standard curve of rAPE1 (equation and R^2^ reported below the table). We then interpolated the densitometry values of huPLTS‐APE1 (p33 + p37) from the PLTs‐WCE onto this curve to obtain the corresponding protein amount. As the PLT number necessary to obtain 1 μg of WCE corresponded to 1, 4 × 10^6^, we calculated the amount of APE1 per single PLT, by dividing the total APE1 content in 1 μg for the number of PLTs needed to obtain 1 μg of extract. Panel (D) PLTs were fractionated into cytosol, microsome and mitochondria compartments by using the mitochondria isolation kit. 50 μg protein *per* fraction were analyzed by western blotting with the reported antibodies. A representative Western blot is shown. Histograms were obtained by densitometry analysis of APE1, CypD, ATP synthase and Tom‐20 in each fraction, expressed as fold increase of their relative WCE densitometry. Values are expressed as mean ± SD (*n* = 4). GAPDH and calnexin were used as marker of cytosol and microsome fraction, respectively. GraphPad V.9.0.0 (Prism) software was used for all statistical analyses, and the mean ± SD values were reported for all data. Panel (E) Characteristics and number of donors enrolled in this study. *Bottom panel*, a representative western blotting analysis of APE1 in PLT‐WCE (50 μg) of a subgroup of donors is reported. The same filter was probed with REVERT for total protein loading and 14–3‐3 γ. Numbers indicate different donors. Panel (F) Graph reporting the normalized APE1 expression in the entire human blood donor cohort. Each bar represents the mean ± SD of the ratio of APE1 on 14–3‐3 γ densitometry values, performed in technical duplicate. Numbers indicate different donors. The color bar indicates different APE1 expression based on 25^th^ percentile above the median (i.e., 0.4), and 25^th^ percentile below the median (i.e., 0.2) (see violin plot). The horizontal broken lines represent the 25^th^ and 75^th^ percentile. Values above the 75^th^ percentile pertain to h‐APE1 donor, values below 25^th^ percentile pertain to l‐APE1 donors. Donor of each one of the two groups (*n* = 8) were pooled for further analyses. Black column: h‐APE1; white column: l‐APE1; gray column: Intermediate APE1 expression. The gray columns were not considered for further analyses. APE1 CVg % of the whole cohort and of h‐APE1 and l‐APE1 groups is reported in the table. CV_g_ was calculated: CVg = $\left(\mathrm{SD}/X\right)$ × 100, where SD represents Standard Deviation of APE1 mean values of each individual, and X is the mean value of APE1 expression in the entire cohort. *Right panel*, Western blotting of APE1 expression in l‐APE1 and h‐APE1 groups (50 μg). REVERT was used as total protein staining and 14–3‐3 γ as normalizer. A representative Western blotting is shown. PLTs: Platelets; WCE: Whole PLT extracts; CypD: Anti‐Cyclophilin D; rAPE1: Recombinant APE1; h‐APE1: High APE1 expression; l‐APE1: Low APE1 expression; CVg: Coefficient of interindividual variability.

To determine the APE1 amount in huPLTs, we used an indirect quantification method based on the densitometry expression of recombinant purified APE1 (rAPE1) and randomized pooled donors‐PLTs lysate, after Western blotting analysis with an anti‐APE1 antibody, which specifically detected both p37 and p33 isoforms (Figure 1C). Results indicated that the amount of APE1 was 6,84 fmol per μg of PLTs lysate, that corresponded to 4,88 × 10^−6^ fmol *per* each PLTs (Figure 1C).

Then, we analyzed the expression of APE1 in huPLTs compartments in randomized subjects (*n* = 4). To this aim, we used specific markers—GAPDH for cytosol, calnexin for microsome fractions, whereas different antibodies were utilized to test the function (ATP synthase, cyclophilin D‐CypD) and integrity (Tom‐20) of isolated mitochondria. Minimal cross‐contamination between mitochondrial markers in the cytosol and non‐mitochondrial markers in the mitochondrial fraction confirmed the high purity and homogeneity of the samples (Figure 1D). APE1 was predominantly distributed in fractions containing mitochondrial markers, clearly exceeding potential mitochondrial contamination (Figure 1D, histograms).

We next tested the APE1 expression variability in the heterogeneous healthy population (*n* = 28), whose characteristics are reported in Figure 1E (Table). Importantly, we chose as normalizer 14‐3‐3 *γ*, a regulator of phosphorylation‐dependent signaling, adhesion, and activation, highly expressed in huPLTs, previously reported in a large cohort as the least variable protein with a coefficient of total variation (CV_tot_) of 9.3% [14], thus representing the best normalizer for biomarker quantification. All samples tested for protein content (REVERT) indicated a similar protein loading, as shown in a population subset (Figure 1E). However, different APE1 expression levels, represented by different ratios of densitometry of APE1 on 14‐3‐3 γ were detected (Figure 1F). In the entire cohort, no statistical differences were found between APE1 expression and donor characteristics (age, PLT count, total cholesterol, triglycerides, sex, and blood group) with Stata 19 (data not shown).

The violin plot representation of the results indicated two groups expressing high (h‐APE1) or low‐APE1 (l‐APE1) (Figure 1F). This led us to group the donors into two pools (*n* = 8 each), based on 25^th^ percentile above or below of the median of APE1 expression in the h‐APE1 and l‐APE1 groups (Figure 1F). Next, we evaluated the extent of APE1 variation between individuals, by means of the coefficient of interindividual variability (CVg) and found that it was approximately 37% in the cohort, whereas 17% in h‐APE1 and 20% in l‐APE1 (Figure 1F). For comparison, previous studies indicated that a typical PLTs protein spot can vary between different individuals by about 18% [14], [15].

To confirm this result, we checked h‐APE1 and l‐APE1 groups for APE1 expression. Accordingly, the results showed a higher amount of APE1 in the h‐APE1 versus l‐APE1 pools, independent of mitochondria CypD levels (Figure 1F, *right panel*), thereby indicating that the APE1 high expression in the h‐APE1 group was independent of the mitochondria amount.

Then, the functionality of l‐APE1 and h‐APE1 was tested through enzymatic activity assay on canonical (ds_dF:dC) and noncanonical substrates (ds_rF:dC and ds_d8oxoG:dC). To this aim, we used tetrahydrofuran (THF) substrate, the closest structural analog to naturally occurring AP‐sites with high stability and specificity for APE1 [16]. Dose–response cleavage assay showed significant cleavage differences between l‐APE1 versus h‐APE1 groups, being a significantly higher enzymatic activity present in the h‐APE1 pool (Figure 2A). To further confirm this result, we performed comparison analysis of the endonuclease assays with the individual donors of l‐APE1 and h‐APE1 groups, and found that APE1 levels correlated with cleavage activity (Figure 2B). Continuous correlation analysis performed on the same samples indicated that APE1 levels correlated with cleavage activity (Pearson's *r* = 0.6582) (Figure 2C). To exclude the role of unknown endonuclease(s) activities in the preparations, we used compound #3, a specific inhibitor of APE1 endonuclease activity [16]. Compound #3 successfully inhibited the enzymatic activity of both pools (Figure 2A, lanes 5 and 9), thus indicating that the detected enzymatic activity of our samples is dependent on APE1.

**FIGURE 2:**
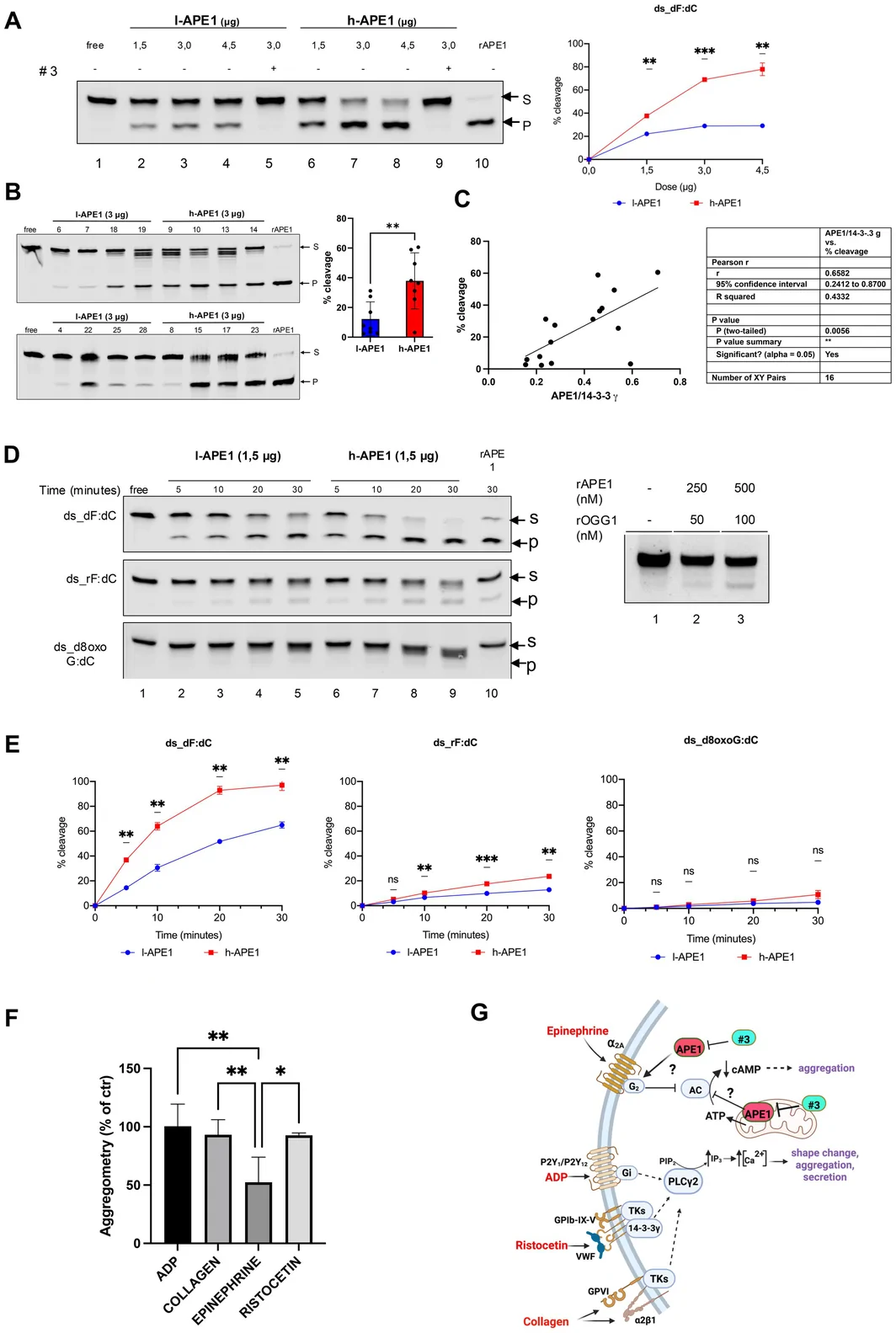
l‐APE1, h‐APE1donor enzymatic activity on dsDNA substrates, and APE1 role in PLT aggregation. Panel (A) Representative denaturing polyacrylamide gel of different doses (μg) of l‐APE1 and h‐APE1 pools on the 25 nM canonical abasic dsDNA substrate (ds_dF:DC). Double‐stranded 25‐mers were generated by annealing 150 pmol of complementary ss_dC oligonucleotide (5′‐GTTCAGGCCTAACACTACCGGATCC‐3′) with 100 pmol of IRDye fluorophore labeled ssDNA oligonucleotide (5′‐GGATCCGGTAGTGTXTAGGCCTGAAC‐3′), containing either a tetrahydrofuran abasic deoxyribonucleotide (ss_dF) at position X. The “free” sample represents the control without protein (lane 1). On lane 10, 0,125 fmol of recombinant APE1 (rAPE1) were incubated with the substrate as positive control. As specificity control, 1 μM compound #3 was added to the reaction (lane 5, lane 9). (On the right, the substrate and the product bands are indicated by two arrows Right panel) Relative graph depicting the dose (expressed in μg) and percentage of cleavage (%) on the x‐ and y‐ axis, respectively. Data are expressed as mean ± SD of two independent technical replicates. Panel (B) Representative denaturing polyacrylamide gels showing APE1 activity on ds_dF:DC substrate in individual donor samples (3 μg). All the reactions were carried out at 37°C for 10 min. Numbers indicate different donors as reported in Figure 1F. The histogram in the inset of this panel shows the variability of APE1 activity among donors within l‐APE1 and h‐APE1 pools. Panel (C) Correlation plot and relative statistical analysis of APE1 levels and % of cleavage. APE1 levels of the donors corresponding to the number above the gels with the corresponding % cleavage evaluated by densitometry of the reported bands in Figure 2B were plotted and analyzed with Prism 9.0. Panel (D) Representative denaturing polyacrylamide gels of time‐course kinetics of l‐APE1 and h‐APE1 (1,5 μg) incubated with the canonical abasic dsDNA substrate (ds_dF:DC) and two noncanonical substrates containing tetrahydrofuran abasic ribonucleotide (ds_rF:DC) and 8‐oxo‐7,8‐dihydro‐2′‐deoxyguanosine (ds_d8oxoG:DC). On the right, positive controls of the assay on ds_d8oxoG: DC oligonucleotide, using recombinant purified APE1 and OGG1, were used. The visible reduction in the substrate band of ds_d8oxoG: DC and its different migration pattern at higher time points could represent exonuclease activity on the substrate. Panel (E) Relative graphs illustrating the time‐course kinetics activity of two PLTS pools with l‐APE1 and h‐APE1 on ds_dF:DC, ds_rF:DC, and ds_d8oxoG: DC. Data are expressed as mean ± SD of two independent technical replicates. Statistical significance between the two groups was analyzed using Student's *t*‐test. **p ≤ 0,01; ***p ≤ 0,001. Panel (F) Aggregometry test. Aggregometry was performed by LTA (Light Transmission Aggregometry). PRP (250 μL) was incubated with compound #3 (10 μM) or DMSO for 15 min at room temperature, followed by incubation with agonists (ADP 2 × 10^−5^ M; collagen 0, 19 mg/mL; epinephrine 1 × 10^−3^ M; ristocetin cofactor 1,5 mg/mL). Histograms represent the final aggregation values for each agonist expressed as % of relative DMSO control. Statistics was performed using one‐way analysis of variance (ANOVA), followed by a posttest analysis Tukey's multiple comparisons test. *p ≤ 0,05; **p ≤ 0,01 (*n* = 5). Panel (G) (Schematic representation of agonists pathways of panel F) in PLT, with the hypothetical role of APE1 in the epinephrine pathway. Our results, showing a significantly reduced aggregation only in the epinephrine‐stimulating condition, raise the hypothesis of a supporting role of APE1 in the adenylate cyclase pathway leading to aggregation. However, at present we could not exclude any additional unknown role of APE1 in the observed effect, also associated with the epinephrine receptor activation itself. We can envision a role for APE1 in blocking the adenylate cyclase pathway and/or positively regulating the a_2A_ receptor. When both these functions are impaired by compound #3, it is conceivable that an increase in cAMP levels leads to diminished platelet aggregation, as observed. Created with Biorender.com.

We also tested l‐APE1 and h‐APE1 groups' enzymatic activities on noncanonical DNA substrates, as ds_rF:dC and ds_d8oxoG:dC [16], with time‐course experiments. In line with the dose–response data, APE1 from the h‐APE1 group was competent to process the canonical substrate ds_dF:dC in a time‐dependent manner with a higher efficiency than the l‐APE1 group (Figure 2D), and noncanonical substrates were cleaved far less by both pools, as expected [16]. The cleavage activity difference between l‐APE1 and h‐APE1 was maintained also on both noncanonical substrates (Figure 2E, middle and right panels). A barely detectable endonuclease activity was registered on ds_d8oxoG:dC, suggesting the absence of any significant glycosylase activity in PLTs (Figure 2D, right panel). Altogether, these data might indicate the ability of APE1‐huPLTs to repair mtDNA damage. Future studies will address this important aspect.

Finally, to highlight the biological relevance of our findings, we examined the role of APE1 in PLT aggregation. To this end, PRP from independent donors (*n* = 5) was subjected to APE1 inhibition with compound #3, followed by incubation with different agonists (ADP, ristocetin cofactor, epinephrine, and collagen) [17]. Results showed a significantly reduced aggregation only in the epinephrine condition (Figure 2F), raising the hypothesis of a specific APE1 involvement in the adenylate cyclase pathway or in a positive α_2A_ receptor regulation (Figure 2G). When these functions are impaired by compound #3, it is conceivable that an increase in cAMP levels may lead to diminished platelet aggregation, as observed. This model is in line with the described role of APE1 in mitochondrial respiration and ATP production in tumor cell lines [18], and discloses APE1's involvement in ATP production in PLTs. Further studies using additional experimental models and other APE1 inhibitors will be necessary to explore this important topic.

Collectively, this initial study describes, for the first time, the expression, amount, and activity of APE1, and its functional role in epinephrine‐induced aggregation in huPLTs, and may pave the way to a population‐based reference interval range of APE1 in healthy subjects, to be estimated in a larger donor cohort. We can speculate that variations in APE1‐huPLTs amount and endonuclease activity could reflect mitochondrial impairment resulting in mtDNA damage accumulation, as previously described in early brain injury context, where APE1 expression significantly decreased alongside increased mitochondrial DNA damage [19]. Consequently, APE1 quantities outside an established reference range might readily unveil mitochondrial dysfunction in PLT activation properties.

## Author Contributions

**Isabella Parolini** designed the study, carried out part of the experiments, performed the analysis, interpreted the data, and wrote the manuscript. **Alessia Bellina** and **Evgeniia A. Diatlova** carried out the enzymatic activity assays and the relative analysis and contributed to manuscript editing and figure preparation. **Elisabetta Fontanini** and **Giovanni Barillari** were responsible for the donor cohort recruitment, PLTs isolation and PLTs aggregation experiments. **Giada Carpi** performed FACS analyses. **Antonio P. Beltrami** interpreted FACS analyses results and contributed to the implementation of the research plan. **Giovanna Lippe** designed and interpreted the results on APE1 mitochondria distribution. **Miriam Isola** performed the statistical analysis of donor cohort characteristics. **Gianluca Tell** designed the overall research plan, contributed to the analysis of the results, the final writing and editing of the manuscript, and he was also responsible for funding acquisition. All authors read and approved the final version of the manuscript submitted for publication.

## Funding

The research leading to these results has been supported by a grant from MUR‐PRIN2022 (grant#20224F7P9Y) to G.T and from AIRC under “IG 2024‐ID. 30399 project—P.I. Tell Gianluca.”

## Conflicts of Interest

The authors declare no conflicts of interest.

## Data Availability

All study data are included in the article. The raw data underlying this article are available in Open Science Framework (OSF), at https://osf.io/qtbzr/overview.

## References

[1] A. Ajanel , R. A. Campbell , and F. Denorme , “Platelet Mitochondria: The Mighty Few,” Current Opinion in Hematology 30 (2023): 167, 10.1097/MOH.0000000000000772. 37459354 PMC10529105

[2] J. Smeitink , L. van den Heuvel , and S. DiMauro , “The Genetics and Pathology of Oxidative Phosphorylation,” Nature Reviews. Genetics 2 (2001): 342–352, 10.1038/35072063. 11331900

[3] B. Li , H. Ming , S. Qin , et al., “Redox Regulation: Mechanisms, Biology and Therapeutic Targets in Diseases,” Signal Transduction and Targeted Therapy 10 (2025): 72, 10.1038/s41392-024-02095-6. 40050273 PMC11885647

[4] C. M. Gustafsson , M. Falkenberg , and N.‐G. Larsson , “Maintenance and Expression of Mammalian Mitochondrial DNA,” Annual Review of Biochemistry 85 (2016): 133–160, 10.1146/annurev-biochem-060815-014402. 27023847

[5] H. Melchinger , K. Jain , T. Tyagi , and J. Hwa , “Role of Platelet Mitochondria: Life in a Nucleus‐Free Zone,” Frontiers in Cardiovascular Medicine 6 (2019): 153, 10.3389/fcvm.2019.00153. 31737646 PMC6828734

[6] S. Pickles , P. Vigié , and R. J. Youle , “Mitophagy and Quality Control Mechanisms in Mitochondrial Maintenance,” Current Biology 28 (2018): R170–R185, 10.1016/j.cub.2018.01.004. 29462587 PMC7255410

[7] G. A. Fontana and H. L. Gahlon , “Mechanisms of Replication and Repair in Mitochondrial DNA Deletion Formation,” Nucleic Acids Research 48 (2020): 11244–11258, 10.1093/nar/gkaa804. 33021629 PMC7672454

[8] L. Lirussi , G. Antoniali , P. L. Scognamiglio , et al., “Cleavage of the APE1 N‐Terminal Domain in Acute Myeloid Leukemia Cells Is Associated With Proteasomal Activity,” Biomolecules 10 (2020): 531, 10.3390/biom10040531. 32244430 PMC7226146

[9] G. Tell , F. Quadrifoglio , C. Tiribelli , and M. R. Kelley , “The Many Functions of APE1/Ref‐1: Not Only a DNA Repair Enzyme,” Antioxidants & Redox Signaling 11 (2009): 601–620, 10.1089/ars.2008.2194. 18976116 PMC2811080

[10] M. Li , Z. Zhong , J. Zhu , et al., “Identification and Characterization of Mitochondrial Targeting Sequence of Human Apurinic/Apyrimidinic Endonuclease 1,” Journal of Biological Chemistry 285 (2010): 14871–14881, 10.1074/jbc.M109.069591. 20231292 PMC2865269

[11] A. Barchiesi , V. Bazzani , A. Jabczynska , et al., “DNA Repair Protein APE1 Degrades Dysfunctional Abasic mRNA in Mitochondria Affecting Oxidative Phosphorylation,” Journal of Molecular Biology 433 (2021): 167125, 10.1016/j.jmb.2021.167125. 34224750

[12] Y. Ding , X. Gui , X. Chu , et al., “MTH1 Protects Platelet Mitochondria From Oxidative Damage and Regulates Platelet Function and Thrombosis,” Nature Communications 14 (2023): 4829, 10.1038/s41467-023-40600-7. PMC10415391 37563135

[13] A. Lador , D. Leshem‐Lev , G. Spectre , A. Abelow , R. Kornowski , and E. Lev , “Characterization of Surface Antigens of Reticulated Immature Platelets,” Journal of Thrombosis and Thrombolysis 44 (2017): 291–297, 10.1007/s11239-017-1533-x. 28785922

[14] R. Baumgartner , E. Umlauf , M. Veitinger , et al., “Identification and Validation of Platelet Low Biological Variation Proteins, Superior to GAPDH, Actin and Tubulin, as Tools in Clinical Proteomics,” Journal of Proteomics 94 (2013): 540–551, 10.1016/j.jprot.2013.10.015. 24284060

[15] W. Winkler , M. Zellner , M. Diestinger , et al., “Biological Variation of the Platelet Proteome in the Elderly Population and Its Implication for Biomarker Research,” Molecular & Cellular Proteomics 7 (2008): 193–203, 10.1074/mcp.M700137-MCP200. 17962630

[16] M. C. Malfatti , S. Balachander , G. Antoniali , et al., “Abasic and Oxidized Ribonucleotides Embedded in DNA Are Processed by Human APE1 and Not by RNase H2,” Nucleic Acids Research 45 (2017): 11193–11212, 10.1093/nar/gkx723. 28977421 PMC5737539

[17] P. A. Wayne , Platelet Function Testing by Aggregometry; Approved Guideline.” CLSI documentH58‐A (Clinical Laboratory Standards Institute, 2008).

[18] M. Codrich , M. Comelli , M. C. Malfatti , et al., “Inhibition of APE1‐Endonuclease Activity Affects Cell Metabolism in Colon Cancer Cells via a p53‐Dependent Pathway,” DNA Repair 82 (2019): 102675, 10.1016/j.dnarep.2019.102675. 31450087 PMC7092503

[19] K. Dai , Z. Wang , B. Gao , et al., “APE1 Regulates Mitochondrial DNA Damage Repair After Experimental Subarachnoid Haemorrhage in Vivo and in Vitro,” Stroke Vasc Neurol 9 (2024): 230–242, 10.1136/svn-2023-002524. 37612054 PMC11221324

